# Predation vs. Parasitism: A Case Study of Indigenous Co‐Stewardship and Science Co‐Production to Measure Temporal Shifts in Moose Mortality on Ancestral Lands of the Grand Portage Ojibwe

**DOI:** 10.1002/ece3.71003

**Published:** 2026-01-25

**Authors:** Tyler J. Garwood, William J. Severud, Steve K. Windels, Arno Wünschmann, Edmund J. Isaac, Anibal G. Armien, Seth A. Moore, Tiffany M. Wolf

**Affiliations:** ^1^ Veterinary Population Medicine, College of Veterinary Medicine University of Minnesota‐Twin Cities St. Paul Minnesota USA; ^2^ Department of Natural Resource Management South Dakota State University Brookings South Dakota USA; ^3^ Voyageurs National Park International Falls Minnesota USA; ^4^ Veterinary Population Medicine, Veterinary Diagnostic Laboratory, College of Veterinary Medicine University of Minnesota‐Twin Cities St. Paul Minnesota USA; ^5^ Department of Biology and Environment Grand Portage Band of Lake Superior Chippewa Grand Portage Minnesota USA; ^6^ California Animal Health and Food Safety Laboratory System, School of Veterinary Medicine University of California‐Davis Davis California USA

**Keywords:** brainworm, cause‐specific mortality, disease ecology, indigenous co‐stewardship, meningeal worm, predation, survival

## Abstract

Maintaining subsistence species on hunting lands is essential to the food security and cultural preservation/flourishing of Indigenous peoples that rely on traditional foods. In northern North America, moose play a central role in subsistence, cultural, and stewardship practices but are declining in many parts of their range. Moose ( *Alces alces* ) in Minnesota are a threatened population that is integral to the lifeways of the Lake Superior Chippewa. This study, led by the Grand Portage Band, examines the shifting causes of adult moose mortality between 2010 and 2022 on the Grand Portage Indian Reservation and Voyageurs National Park. These efforts, rooted in principles of Indigenous sovereignty and co‐stewardship, seek to sustain this vital species on ancestral lands. We observed that the relative importance of mortality causes varied over time, with *Parelaphostrongylus tenuis* and other health‐related factors driving mortality during the initial decline period (2010–2014), while predation became a leading cause of mortality and quadrupled in probability during the stabilization period (2015–2022). Using a Bayesian framework, we integrated multiple contributing factors to accurately estimate cause‐specific mortality probabilities and survival rates. The findings underscore the necessity for adaptive management strategies that address both parasitism and predation pressures to recover moose populations to pre‐decline levels. Moreover, this study exemplifies how a long‐term, Indigenous‐led wildlife collaring and monitoring program is critical to capturing these dynamics and supporting the Grand Portage Band's ongoing stewardship. This research advances our understanding of moose mortality in a vulnerable population and reinforces the importance of Indigenous leadership in wildlife management and scientific co‐production. By integrating traditional ecological knowledge found in Tribal‐governmental planning documents with contemporary science led by their Natural Resources Management department, the Grand Portage Band is ensuring that moose remain a resilient and enduring part of their cultural and subsistence practices, thus contributing to the broader framework of Indigenous co‐stewardship.

## Graphical abstract

This study, led by the Grand Portage Band of Lake Superior Chippewa, tracks survival and examines causes of adult moose mortality from 2010 to 2022 on the Grand Portage Indian Reservation and in Voyageurs National Park. Using a Bayesian framework, we integrated multiple contributing factors to accurately estimate cause‐specific mortality probabilities and survival rates, and found that *P. tenuis* and other health issues were leading causes of death during a period of moose population decline, whereas predation was a leading cause of death when the population trajectory stabilized but failed to recover. The findings underscore the necessity for adaptive management strategies that address both parasitism and predation pressures to ensure the long‐term sustainability of moose populations and exemplify how long‐term, Indigenous‐led monitoring programs are critical to capturing these dynamics.

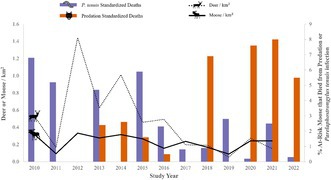

## 1 Introduction

The food security and cultural flourishing of many Indigenous peoples benefit from harvestable subsistence species populations on their contemporary hunting lands. Indigenous people living off‐reservation, and therefore, often away from hunting lands and subsistence species, can be four times more likely to be food‐insecure than those living on reservation (Priadka et al. 2022; Willows et al. 2008). The loss or dispossession of traditional resources can also disrupt the transmission of cultural knowledge between generations (Tobias and Richmond 2014), whereas the maintenance of subsistence food traditions can be a source of spiritual healing and strengthening (Marquina‐Márquez et al. 2016). Hence, conserving traditional subsistence species is a primary goal of many Indigenous nations, despite the challenges posed by climate change and other environmental stressors (Lynn et al. 2013).

Indigenous co‐stewardship can be useful to address subsistence species declines that impact Indigenous lifeways. Co‐stewardship emphasizes a collaborative approach to natural resource management where Indigenous knowledge systems, values, and governance structures are central to decision‐making processes (Moore, Severud, et al. 2024; Moore, Wolf, et al. 2024). Central tenets of Indigenous co‐stewardship are recognition of Indigenous Sovereignty, shared responsibility, cultural/ecological respect, inclusive decision‐making, resource sharing, capacity building, adaptive management, and sustainability. For Indigenous nations, it is therefore an exercise of sovereignty and a necessary step to ensure that their lifeways, and those of future generations, are preserved (Popp et al. 2019). For collaborating scientists and institutions, adhering to the tenets of co‐stewardship creates an equitable environment that allows for new perspectives, insights, datasets, and techniques that lift the fields of ecology and conservation as a whole.

The decline of North American moose ( *Alces alces* , *mooz*‐singular, *moozoog*‐plural Ojibwe language) populations along their southern range boundary threatens biodiversity, ecological interactions, regional persistence, and thereby the cultural and physical survival of many Indigenous peoples (Ross and Mason 2020; Bump et al. 2017; Mathisen and Skarpe 2011). Indigenous cultures have subsisted on moose for thousands of years and continue to harvest moose for subsistence (Priadka et al. 2022; Natcher et al. 2021). Furthermore, moose are symbolic of cultural heritage and are deeply embedded in identity and responsibilities as Indigenous stewards of the land (Popp et al. 2020; LeBlanc et al. 2011). The survival of moose is, therefore, intricately linked to the continuation of subsistence lifeways and cultural vitality.

To address Minnesota's moose population decline from ~8000 to ~4000 individuals (~50%) during 2005–2013 and the ensuing lack of recovery (Giudice 2023), the Grand Portage Anishnabeg (original people, Ojibwe language; or Band of Lake Superior Chippewa) took a leading role in the research and management efforts to understand and address contributors to the decline and lack of recovery using the co‐stewardship framework. The Grand Portage Band is a sovereign Indigenous nation that holds profound responsibilities and rights over the lands and wildlife within their ancestral territories, as indicated by inherent Indigenous sovereignty and federally recognized treaty agreements (Pevar 2012; Bauerkemper 2015; Treaty with the Chippewa 1854). To uphold these responsibilities, the Grand Portage Band of Lake Superior Chippewa has led and conducted long‐term ecosystem health research since 2010, which focused on the perpetuation of moose as a vital subsistence species and engaged federal, state, and academic collaborators in the co‐stewardship framework. The long‐term research program was catalyzed by two guiding Tribal Governmental documents that incorporated Traditional Ecological Knowledge through development with community perspectives. Both governmental guiding documents, the Grand Portage Integrated Resource Management Plan (Novitsky 1987) and the Grand Portage Strategic Plan to Adapt to Climate Change (Moore, Wolf, and Travis 2015) identify moose as the primary terrestrial subsistence species for Tribal and Ceded lands and direct the Grand Portage Natural Resources Management Agency to focus on both biological and landscape management activities for the purpose of seventh generation planning to maintain viable and harvestable populations of moose on the landscape (Hoagland and Albert 2023).

To follow through with these plans, the Grand Portage Natural Resources Management agency secured more than $15 M USD to develop and lead moose research in a co‐stewardship framework with a particular commitment to data sovereignty (Walter et al. 2021; Moore, Wolf, and Travis 2015). This effort resulted in over 50 peer‐reviewed publications on moose, ranging from health and physiology (Wolf et al. 2021; Ienello et al. 2021; Fountain‐Jones et al. 2020; Struck et al. 2023; Verma et al. 2015), parasite and disease transmission (Severud, Giguere, et al. 2023; Severud, Kautz, et al. 2023; Garwood et al. 2023; Verant et al. 2022), habitat and landscape use (Van de Vuurst et al. 2022; Oliveira‐Santos et al. 2021; Orning et al. 2020; Romanski et al. 2020; Street et al. 2016), predator interactions and associated diet (Chenaux‐Ibrahim et al. 2024; Moore, Wolf, et al. 2024; Sovie et al. 2023; Hervey et al. 2021), and population estimation (Severud et al. 2022; McMahon et al. 2021). Since this effort is a direct response to the leadership of the Tribal government, it thereby originates from modern Indigenous ecological knowledge and illustrates Indigenous science co‐production.

Chief among the Grand Portage Band's yet‐unanswered research aims is to understand the causes of mortality among adult and yearling moose in northeastern Minnesota, which is essential for developing management strategies that will allow the moose population to recover and continue to be a reliable subsistence resource. Understanding the drivers of adult mortality rates is of particular interest because they can disproportionately affect population growth rates (Schaub 2009; Eberhardt 2002), and correctly identifying temporal shifts is integral to population management (Cristescu et al. 2022). Despite their importance, most cause‐specific mortality (CSM) studies remain temporally short and use methodologically limited estimation techniques (Hill et al. 2019; Heisey and Patterson 2006). A short‐duration adult mortality assessment was previously conducted in northeastern Minnesota but could not investigate CSM changes over time (Carstensen et al. 2017).

Parasitism is a key contributor to adult moose mortality globally and locally (Niedzialkowska et al. 2022; Lankester 2010). *Parelaphostrongylus tenuis* is a nematode that asymptomatically infects white‐tailed deer ( *Odocoileus virginianus* , hereafter “deer”) but causes neurological damage when it aberrantly infects other cervids. The parasite recently expanded its range, driving moose declines across central and eastern North America (Lankester 2010). *Parelaphostrongylus tenuis* incidence in moose correlates with deer density; although the exact relationship remains debated, 4 deer/km^2^ is suggested as a plausible number for reducing transmission to moose (McGraw et al. 2021; Minnesota Department of Natural Resources 2011). Winter tick ( *Dermacentor albipictus* , hereafter “tick”) epizootics, where many ticks feeding on moose cause anemia, are also associated with moose declines (Musante et al. 2010). These declines are often short‐lived and in high‐density moose populations (Lankester 2010). Unlike other moose parasites, ticks and *P. tenuis* cause significant morbidity and mortality at the population level (Jones et al. 2019; Carstensen et al. 2017; Wünschmann et al. 2015; Lankester 2010).

The extent to which wolves ( *Canis lupus* ) kill healthy, prime‐aged adult moose remains unclear (Mech and Nelson 2013). Wolves target diseased moose, which may be compensatory mortality (Hoy et al. 2022). In northeastern Minnesota, wolf predation (hereafter, “predation”) peaks in spring and winter seasons (Wehr et al. 2024a, 2024b; Van Van den Bosch et al. 2023) and was previously identified as the top cause of adult moose mortality (32% of mortalities; Carstensen et al. 2017). However, 44% of predator‐killed moose had other conditions that could cause mortality ( *P. tenuis* infection, trauma, unidentified health issues). Had those mortalities been attributed to predisposing factors, predation would account for 18% of mortality and be less prevalent than parasitism and other health issues.

Methods assigning a single cause of mortality when there are multiple contributing factors obfuscate population‐level inferences. It can be impossible to accurately assign a single cause to a given death, and doing so can lead to biased estimates of CSM (Walsh et al. 2018). Bayesian methods can incorporate the multifactorial nature of these deaths, via prior predictive probabilities, into a formal CSM modeling framework (Walsh et al. 2018). These methods are rarely used despite improving the clarity and accuracy of CSM estimates (Cristescu et al. 2022; Garwood et al. 2020).

The primary objective of our study was to demonstrate effective Indigenous co‐stewardship principles while investigating the relative importance of adult moose mortality causes in ancestral and current Indigenous‐held lands in Minnesota and provide evidence of their contribution to the population decline and subsequent lack of recovery. Thus, the Tribal natural resources department and collaborators collared yearling and adult moose, tracked survival, and documented mortalities. We calculated survival estimates and used Bayesian methods to integrate cause of death uncertainty into our estimates of CSM estimates and survival. We then compared those metrics in association with the timing of the population's decline, subsequent stabilization, and annual deer density estimates. We hypothesized that, in accounting for other contributing factors at the time of mortality, predation would be less important than previously thought, and the relative importance of different causes would vary temporally and relative to deer population dynamics.

## 2 Methods

### 2.1 Study Area

**FIGURE 1:**
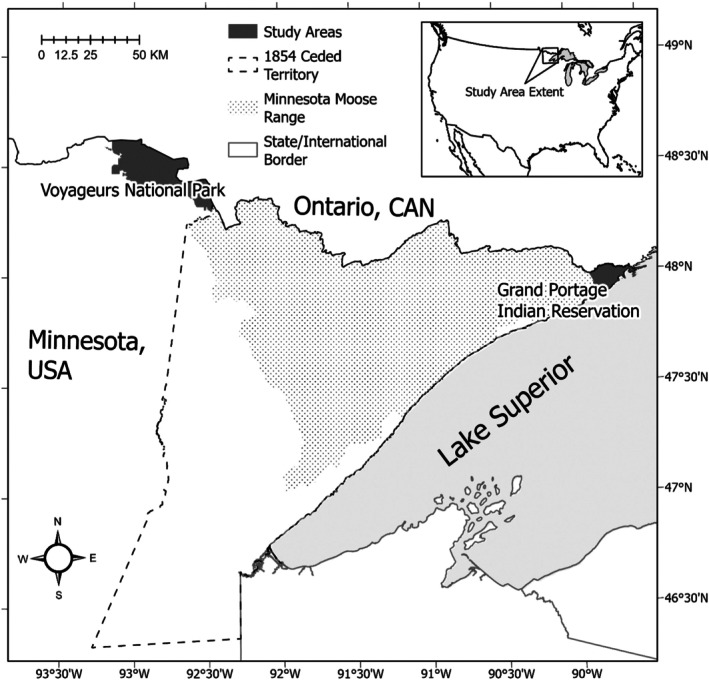
Study areas, current moose range, and the 1854 Ceded Territory in Minnesota, USA. The Grand Portage Band and other Lake Superior Chippewa reserve the right to harvest moose across the 1854 Ceded Territory and co‐manage this important subsistence species with the Minnesota Department of Natural Resources and US federal government agencies.

This study took place on and around Grand Portage Indian Reservation (GPIR) and Voyageurs National Park (VNP), which represent the northeastern and northwestern extent of Minnesota moose range, respectively (Figure 1). Other large mammals in both study areas include wolves, deer, and black bears ( *Ursus americanus* ).

Grand Portage Indian Reservation covers 192 km^2^, bordered by Ontario, Canada, to the north and Lake Superior to the east and south. GPIR is located in the boreal/mixed conifer‐hardwood forest transition zone. Common tree species include sugar maple ( *Acer saccharum* ), paper birch ( *Betula papyrifera* ), white pine ( *Pinus strobus* ), white cedar ( *Thuja occidentalis* ), aspen (*Populus* spp.), balsam fir ( *Abies balsamea* ), and black spruce ( *Picea mariana* ). To the west of the reservation is largely state and federal land, which is part of the 1854 Ceded Territory of Minnesota, where the Grand Portage Band and other Lake Superior Chippewa tribes co‐manage natural resources with the federal and state government. They retain rights to hunt, fish, and gather traditional subsistence species, including moose, on these lands (Thompson 2017).

Voyageurs National Park covers 882 km^2^, bounded in the north by Ontario, Canada, and otherwise surrounded by state, national, and private land. Moose hunting was prohibited within VNP at the time of park establishment and ceased outside the park in 2013. The habitat is southern boreal and Laurentian mixed conifer‐hardwood forested land (61%) and large lakes (39%). Common trees include quaking aspen ( *P. tremuloides* ), paper birch, balsam fir, white spruce ( *P. alba* ), white pine, red pine ( *P. resinosa* ), jack pine ( *P. banksiana* ), and black spruce.

### 2.2 Collaring, Monitoring, Necropsy, and Cause of Death Assignment

We followed the capture/collaring protocol described in Oliveira‐Santos et al. (2021). Briefly, we aerially darted 153 moose between 2010 and 2022 in GPIR and 21 moose between 2010 and 2012 in VNP. Sex and age class were recorded. We fitted moose with global positioning system (GPS) collars that reported a mortality if movement was below a programmed threshold. All capture and handling protocols followed the University of Minnesota (UMN) Institutional Animal Care and Use Committee (protocols 1803‐35736A, 0192A75532, and MWR_VOYA_Moen_Moose_2011A2).

A mortality notification was sent as a cell phone text and email by satellite to investigators when a collared moose had not moved in ~6 h. As soon as possible after receiving a mortality notification (usually within 24 h; Table S1), biologists with field necropsy training located the moose. After recording observational cause of death evidence at the mortality location (e.g., signs of struggle, predator sign), biologists performed a necropsy. Occasionally, due to complex terrain, gaps in GPS data transmission, or scheduling conflicts, dead moose were not recovered prior to the start of autolysis. Although necropsies varied with the severity of carcass autolysis or consumption by predators and scavengers, they consisted of assessing moose body condition (fat content, bone marrow condition, hair loss, ectoparasitism), external and internal trauma (bite marks, broken bones, hemorrhages), and internal organ condition (cysts, parasitism, and other lesions in lungs and liver). Tissues, organs, and blood/serum were shipped to the UMN Veterinary Diagnostic Lab (VDL; St. Paul, MN, USA) for further examination. If available, a tooth was removed postmortem and sectioned for aging (protocol in Boertje et al. 2015; Matson's Laboratory, Manhattan, MT, USA). Our field team additionally examined the GPS point data from 1 week prior to the mortality for movement patterns that could help determine the cause(s) of death. For example, increased localization and reduced spread of points days prior to mortality would provide evidence for a health‐related cause, whereas a large, quick movement followed by localization would provide evidence of a predation event. The findings from the field necropsy and relevant observations of GPS movement data or site investigation were recorded.

To the extent possible, histopathology and other diagnostic tests of biological samples collected at necropsy were performed at the UMN‐VDL. Common tests are described in Wünschmann et al. (2015, 2021). These included histopathology to identify microscopic tissue lesions, such as those induced by *P. tenuis* infection. The findings from the laboratory necropsy and additional tests were compiled into a diagnostic report, including test results, a findings summary, and diagnosis by the case pathologist.

Based on the combined data, we (five field biologists, one veterinarian, two pathologists) assigned a probability for each cause of death classification for each individual. Our cause of death classes were (1) *P. tenuis* infection, (2) other health, (3) tick infestation, (4) predation, and (5) other. *Parelaphostrongylus tenuis* infection was defined by histopathological evidence or antemortem observations of abnormal behavior via direct observation (from the air or on the ground) or GPS data prior to death (e.g., head tilt or circling, atypical GPS movement patterns, such as very localized position for a period of several days prior to death). Other health was defined by health‐related issues that played a significant role in death but were not related to *P. tenuis* or ticks (e.g., poor nutrition, bacterial infections, dystocias). Tick infestation was defined by significant (> 25%) hair loss or patches of broken hair, massive (> 1000) tick numbers, and/or skin crusts over large areas of the body. Mortality caused by ticks was defined by pathologist‐determined anemia and overall cachexia (Valli et al. 2016). Both anemia and cachexia are well‐defined metrics, and the qualitative metrics, such as hair loss and tick numbers, were used as data (Table S1) to validate that ticks caused the anemia and cachexia. Predation was defined by attack signs at the death site (e.g., broken shrubs and heavily disturbed ground cover, wolf prints in snow, blood splatters on nearby vegetation) and/or bite marks and associated hemorrhages. Other was defined as traumatic injury, hunter harvest, or presumed senescence (inferred from animal age and whether the death circumstances would cause mortality in younger individuals). As an example of this process, field biologists might have reported that the carcass was mostly consumed by wolves, there were signs of struggle, the hide lacked signs of abnormal tick load, and the bone marrow had a gelatinous consistency (indicating poor nutrition). The UMN‐VDL pathologists might have additionally reported that the brain lacked histopathological signs of *P. tenuis* . Because predation caused this death, but nutritional compromise played a role, we would assign an 80% probability to “predation” 20% to “other health” and 0% to all other cause classes.

### 2.3 Model Structure

**FIGURE 2:**
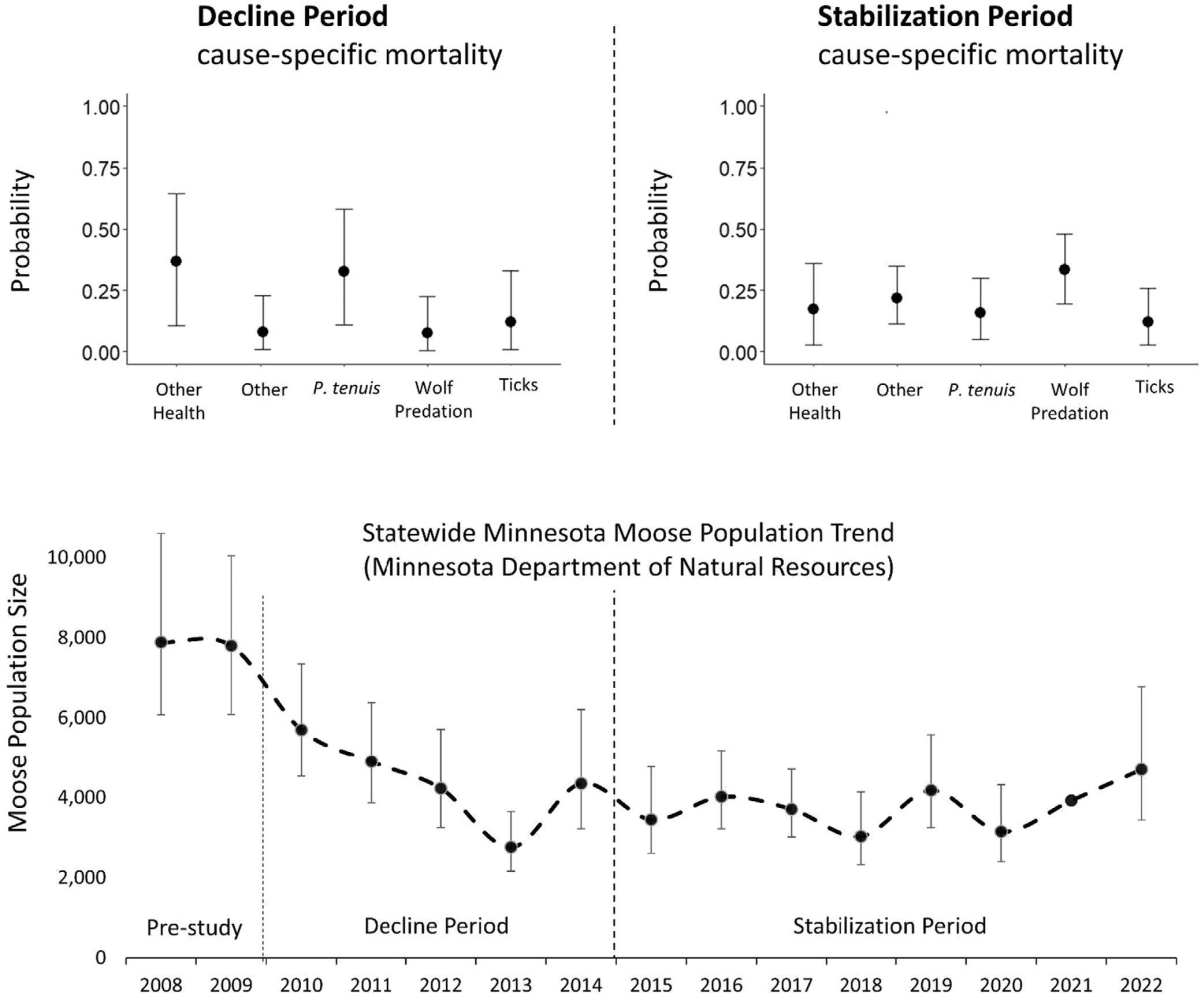
Cause‐specific mortality probability estimates across study areas split into the decline and stabilization periods of the moose population. Statewide moose numbers estimated by the Minnesota Department of Natural Resources are shown for reference (Giudice 2023).

The data on each individual used in our analysis were (1) time of capture (*e*_*i*_), (2) the last week an individual was known to be alive (*r*_*i*_), (3) the week the individual was confirmed dead (*s*_*i*_), and (4) a vector describing the probability that the individual died from each source of mortality, with one mortality being assigned as at least slightly (i.e., 1%) more probable than others. These data were incorporated into a Bayesian two‐component time‐to‐event model, which first modeled the overall hazard of dying irrespective of cause of death, and then, conditional on death, modeled the probability of dying due to various sources of death. We implemented this framework in the Nimble package in R (R Core Team 2023; de Valpine et al. 2017). We analyzed all study years simultaneously, as well as separately in a “decline period” (1 February 2010 to 31 December 2014) and a “stabilization period” (1 January 2015 to 31 December 2022). These were determined a priori based on well‐established and accepted Minnesota moose population trends (Figure 2; Giudice 2023). The decline period was characterized by a 46% decline in the estimated moose population size over 5 years; whereas during the stabilization period, moose populations neither recovered to pre‐decline numbers nor continued declining.

The *e*_*i*_, *r*_*i*_, and *s*_*i*_ were used to estimate survival via the cumulative hazard function, with the individual likelihood contribution being

$$\mathrm{Pr}\;\left(r_{i}\leq T_{i}<s_{i}|T_{i}\geq e_{i}\right)=\exp\;\left(-\int_{e_{i}}^{r_{i}}h\left(t\right)\mathrm{dt}\right)\;\left[1-\exp\;\left(-\int_{r_{i}}^{s_{i}}h\left(t\right)\mathrm{dt}\right)\;\right]$$

where $T_{i}$ is the random time of death and *h(t)* is the instantaneous hazard function. A piecewise constant hazard function was used to approximate the cumulative hazard function such that

$$\int_{r_{i}}^{s_{i}}h\left(t\right)\mathrm{dt}\approx \sum_{r_{i}}^{s_{i}}{Ʌ}_{u}$$

with ${Ʌ}_{u}$ being the unit cumulative hazard for each week ($\int_{r_{i}}^{r_{i}+1}h\left(t\right)\mathrm{dt}$). We modeled survival status as a conditionally independent Bernoulli trial such that

$$y_{\mathrm{ij}}\;\sim \;\text{Bern}\left(\exp\left(-\sum_{k=r_{\mathrm{ij}}}^{s_{\mathrm{ij}}}{Ʌ}_{u}\right)\right)$$

where *s*_*ij*_ is the start of the *j* ^th^ time interval for individual *i* and *r*_*ij*_ is the end of the *j* ^th^ interval for individual *i* (Cross et al. 2015). To clarify this structure, if an individual dies several years into the study, they will contribute to the data likelihood twice; once for the interval when they were alive (“success”: $\exp\left(-\sum_{e_{i}}^{r_{i}}{Ʌ}_{k}\right)$) and once over the interval during which they died (“failure”;$\;1-\exp\left(-\sum_{r_{i}}^{s_{i}}{Ʌ}_{k}\right)$). If an individual survives to the end of the study, they are right‐censored and contribute to the data likelihood once (“success”: $\exp\left(-\sum_{e_{i}}^{r_{i}}{Ʌ}_{k}\right)$). Covariates were incorporated into the log unit cumulative hazard as

$$\ln\left({Ʌ}_{i,u}\right)=\gamma +\beta X_{i,u}+\rho_{u}$$

where γ is the baseline log cumulative hazard rate, β is the vector of coefficients, $X_{i,u}$ is the vector of predictor variables for the *i*th individual for the *u*th week. We specified the covariate effect priors as diffuse (*β*_*x*_ ~ Normal [0, *σ* ^*2*^ = 100]). *ρ*_*u*_ is a smoothing function between weeks (*ρ*_*l*_ ~ Uniform [−0.5, 0.5]) for the first week effect, *ρ*_*u*_ ~ Normal[*ρ*_*u − 1*_, *σ* ^*2*^ = 1/*τ*] for the *u*th week, *τ* = 1/*σ* ^*2*^ (*σ* ^*2*^ ~ Uniform [0,12]; Gelman et al. 2014; Cressie and Wikle 2011). We used weakly informative truncated normal priors on the baseline log unit cumulative hazards and assumed a mean annual survival of 89% and a 95% probability of lying between 20% and 95% (Franzmann and Schwartz 2007; *γ* ~ Normal [−6.1, *σ* ^*2*^ = 0.29] T[−9.5, −2]). We also tested a prior assuming 86% annual survival (*γ* ~ Normal [−5.5, *σ* ^*2*^ = 0.29] T[−9.5, −2]); survival was reduced by less than 2% with this alternative prior.

In the second component of the model, we used the observer‐assigned vector of probabilities of the cause of death to account for uncertainty in cause of death assignments. The theory behind this approach is extensively explained and justified in Walsh et al. (2018). Briefly, an individual's cause of death was modeled as:

$${\text{cause}}_{i,k}\sim \mathrm{Cat}\left(\pi_{k}\right)$$

$${\text{cause}}_{i,k}=\{1\;\text{if}\;{\text{cause}}_{i}=k,\;0\;\text{if otherwise}$$

Covariates were incorporated using a multinomial logistic model for each individual such that:

$$\pi_{i,k}=\frac{\exp\left(\alpha_{k}+\delta_{k}x_{i}\right)}{\sum_{h=1}^{H}\exp\left(\alpha_{h}+\delta_{h}x_{i}\right)}$$

where $\pi_{i,k}$ is the probability of the *k*th cause of death for *i*th individual, $\alpha_{k}$ 2 is the intercept term for the kth cause of death, $\delta_{k}$ is the vector of coefficients for death category *k*, and $x_{i}$ is the vector of predictor variables for the *i*th individual. The reference cause of death was “other”. We specified diffuse covariate and baseline mortality priors as:$\;\left(\alpha_{k}\sim \text{Normal}\;\left[0,\;\sigma^{2}=100\right];\mathrm{\beta x}\sim \mathrm{No}\text{rmal}\;\left[0,\sigma^{2}=100\right]\right).$

Note that we sometimes were uncertain about the “true” cause_*i,k*_, especially in the case where a death was multifactorial. In such a situation, we used misclassification theory (Hoenig et al. 2002) to get a more accurate idea of *cause*_*i,k*_ than if we simply applied our error‐prone assignment. From a misclassification theory perspective, each mortality has two associated random variables: (1) the true fate (*A*_*m*_ *= i*), which is unobserved and (2) the observed but error‐prone fate (*E*_*m*_ *= j*). *E*_*m*_'s must be corrected before being considered representative of cause_*i,k*_. From a practical perspective, we performed this correction via data augmentation (Tanner and Wong 1987) where, for each death, cause_*i,k*_ is drawn at each MCMC iteration (100,000 iterations, 10,000 iteration burn‐in, three chains) from a categorical distribution with parameters set by the vector describing the probability that the individual died from each source of mortality. We checked for convergence with trace plots. When compiled across individuals and mortalities, this provides a more accurate estimate of cause‐specific probabilities for the population than simply assigning a single, error‐prone cause (Walsh et al. 2018).

The full likelihood for the *i*th adult moose is thus:

$$\mathrm{Pr}\;\left(r_{i}\leq T_{i}<s_{i},K=k|T_{i}\geq e_{i}\right)=\exp\;\left(-\sum_{e_{i}}^{r_{i}}{Ʌ}_{i,u}\right)\times \left[1-\exp\;\left(-\sum_{r_{i}}^{s_{i}}{Ʌ}_{i,u}\right)\;\right]\times \pi_{i,k}$$

which allows for staggered entry, interval censoring, and right censoring.

For model selection, we calculated the Watanabe‐Akaike Information Criterion (WAIC; Gelman et al. 2014). We defined the best model as that with the lowest WAIC. We assessed model fits with graphical posterior predictive checks.

### 2.4 Covariates and a Priori Models

We modeled weekly survival rates and CSM while incorporating study site, age class/death age class (yearling [1–2 years old] or adult [2+ years old]; Keech et al. 2011), and sex (Boertje et al. 2020) as covariates. We coded study site, death age class, and sex as binary (VNP =1, GP = 0; Yearlings = 1, Adults = 0; Male = 1, Female = 0) and age class as binary and time varying (Yearlings = 1, Adults = 0). Moose advanced age class on June 1, which is typically the end of the birth pulse in our study area (Severud et al. 2015). Our global model for survival was *γ + β*_study area_ *×* study area_*i*_ *+ β*_sex_ *×* sex_*i*_ *+ β*_age class_[ageclass_*ij*_] *+ ρ*_*j*_., where *β*_study area_ was the study area effect, *β*_sex_ was the sex effect, and β_age class_ was the age class effect. Annual estimates of survival were calculated by ln(−ln($\gamma^{52}))$. Our global model for CSM was$\;\alpha_{k}$ *+ β*_study area_ *×* study area_*ik*_ *+ β*_sex_ *×* sex_*ik*_ *+ β*_death age class_ *×* death age class_*ik*_. *β*_death age class_ was the effect of the age class at death. All covariates were assessed for statistical significance using a 95% confidence level and all parameter estimates are reported with 95% equal‐tailed credible intervals (2.5th and 97.5th percentiles).

### 2.5 Moose and Deer Density Estimates

We performed moose surveys on GPIR following standard local protocols (Giudice 2023). Briefly, we gridded GPIR into evenly spaced 530 m east–west transects. A helicopter flew transects from 2010 to 2022. During flights, we enumerated moose, identified sex and age class, and recorded location. We assessed sex via antlers/pedicles, vulva patches, and nose color, size, and shape. Calves were identified by size, behavior, and association with other moose. Collared moose observed in the survey were identified with GPS collar data post‐survey. We calculated a sightability correction factor by dividing the number of collared moose present by the number of collared moose observed in the survey area. Density estimates were calculated by multiplying the number of moose spotted by the correction factor. This survey expanded from 203 to 399 km^2^ in 2020.

We counted deer during the flights described above but did not adjust for sightability due to limited collared deer numbers. Conservative densities were estimated by dividing the raw number of deer spotted by the survey area.

## 3 Results

### 3.1 Collaring, Monitoring Effort, and Deaths Documented

We collared 174 individuals (52 bulls, 122 cows; 20 yearlings, 154 adults) and observed 70 mortalities between 1 February 2010 and 4 December 2022 (Table 1 for detailed breakdown). The remaining 104 individuals were right‐censored. We responded to 93% of mortalities within 48 h (general condition of carcass and samples collected described in Table S1). During the decline period, we tracked 64 individuals and 20 mortalities. During the stabilization period, we monitored 140 individuals and documented 50 mortalities. Survival data from some individuals were included in both the decline and stabilization period analyses because their lifetime spanned both periods. We knew the death age for 47 individuals (mean = 6.2 years, age range = 1–19).

**TABLE 1:** Summary of the data used to calculate moose cause‐specific mortality probabilities and survival 2010–2022 in northeastern Minnesota.

	Collared			Mortalities		
	Bulls/Cows	Yearlings/Adults	Total	Bulls/Cows	Yearlings/Adults	Total
	**Overall**					
Grand Portage Indian Reservation	45/108	20/133	153	19/41	4/56	60
Voyageurs National Park	7/14	0/21	21	3/7	0/10	10
Total	52/122	20/154	174	22/48	4/66	70
	**Decline**					
Grand Portage Indian Reservation	7/36	3/40	43	3/11	1/13	14
Voyageurs National Park	6/15	0/21	21	2/4	0/6	6
Total	13/51	3/61	64	5/15	1/19	20
	**Stabilization**					
Grand Portage Indian Reservation	38/93	17/114	131	16/30	3/43	46
Voyageurs National Park	2/7	0/9	9	1/3	0/4	4
Total	40/100	17/123	140	17/33	3/47	50

### 3.2 Cause‐Specific Mortality

During the entire study period and across study areas, other health (probability = 25.1%, 95% Credible interval [CI] = 10.5%–41.1%) was the leading cause of death, followed by predation (24.7%, 14.1%–36.6%), *P. tenuis* (20.5%, 9.8%–33.2%), other (17.7%, 9.1%–28.3%), and ticks (12.1%, 3.7%–23.5%). Our best CSM model was $\alpha_{k}$ *+ β*_study area_ *×* study area_*ik*_ *+ β*_sex_ *×* sex_*ik*_ (*w*_*i*_ = 0.419; Table 2). Study area and sex did not have a statistically significant effect on CSM probabilities.

**TABLE 2:** Rankings of all models tested in estimating cause‐specific mortality and survival for adult moose ( *Alces alces* ) during the entire study period (1 February 2010 to 4 December 2022) and split in a “decline period” (1 February 2010 to 31 December 2014) and “stabilization period” (1 January 2015 to 31 December 2022). Ranking is based upon Watanabe‐Akaike Information Criterion (WAIC) and is reported with *w*_*i*_ (WAIC weight). β_study area_ is the effect of the study area, β_sex_ is the effect of sex. For cause‐specific mortality models, β_death age class_ is the effect of age class at death (yearling vs. adult) and $\alpha_{k}$ is the intercept term. For the survival models, β_age class_ is the effect of age class, γ is baseline log unit cumulative hazard rate, and ρ_j_ is the effect of a given week (j) with a random walk prior for temporal smoothing across estimates.

Overall			Decline			Stabilization		
Model covariates	WAIC	*w*_*i*_	Model covariates	WAIC	*w*_*i*_	Model covariates	WAIC	*w*_*i*_
**Cause‐specific mortality**								
$\alpha_{k}$, β_study area_, β_sex_	1171.0	0.419	$\alpha_{k}$, β_death age class_	355.0	0.302	$\alpha_{k}$, β_death age class_, β_sex_	813.5	0.596
$\alpha_{k}$, β_death age class_, β_sex_	1171.1	0.397	$\alpha_{k}$, β_death age class_, β_sex_	355.6	0.220	$\alpha_{k}$, β_study area_, β_death age class_, β_sex_	814.6	0.344
$\alpha_{k}$, β_study area_, β_death age class_, β_sex_	1173.3	0.128	$\alpha_{k}$, β_sex_	356.3	0.157	$\alpha_{k}$, β_sex_	819.1	0.036
$\alpha_{k}$, β_sex_	1175.0	0.056	$\alpha_{k}$, β_study area_, β_death age class_, β_sex_	356.9	0.117	$\alpha_{k}$, β_study area_, β_sex_	819.9	0.024
$\alpha_{k}$	1189.4	0.000	$\alpha_{k}$	357.9	0.071	$\alpha_{k}$, β_study area_, β_death age class_	831.9	0.000
$\alpha_{k}$, β_study area_, β_death age class_	1189.7	0.000	$\alpha_{k}$, β_study area_, β_death age class_	358.3	0.058	$\alpha_{k}$, β_death age class_	831.9	0.000
$\alpha_{k}$, β_study area_	1190.3	0.000	$\alpha_{k}$, β_study area_	358.9	0.043	$\alpha_{k}$	833.1	0.000
$\alpha_{k}$, β_death age class_	1190.7	0.000	$\alpha_{k}$, β_study area_, β_sex_	359.4	0.033	$\alpha_{k}$, β_study area_	833.2	0.000
**Survival models**								
γ, β_study area_, β_age class_, ρ_j_	1187.7	0.328	γ, β_study area_, ρ_j_	356.5	0.443	γ, β_study area_, β_age class_, ρ_j_	830.7	0.324
γ, β_age class_, ρ_j_	1188.2	0.256	γ, ρ_j_	357.9	0.220	γ, β_age class_, ρ_j_	832.3	0.143
γ, ρ_j_	1189.4	0.140	γ, β_study area_, β_sex_, ρ_j_	359.1	0.121	γ, β_study area_, ρ_j_	832.5	0.132
γ, β_age class_, β_sex_, ρ_j_	1189.5	0.133	γ, β_age class_, ρ_j_	359.7	0.089	γ, ρ_j_	833.1	0.095
γ, β_study area_, β_sex_, ρ_j_	1191.3	0.054	γ, β_study area_, β_age class_, ρ_j_	360.8	0.052	γ, β_age class_, β_sex_, ρ_j_	833.1	0.094
γ, β_sex_, ρ_j_	1191.5	0.049	γ, β_sex_, ρ_j_	360.8	0.052	γ, β_study area_, β_sex_, ρ_j_	833.3	0.086
γ, β_study area_, ρ_j_	1192.0	0.038	γ, β_age class_, β_sex_, ρ_j_	362.6	0.021	γ, β_sex_, ρ_j_	833.6	0.074
γ, β_study area_, β_age class_, β_sex_, ρ_j_	1199.6	0.001	γ, β_study area_, β_age class_, β_sex_, ρ_j_	367.7	0.002	γ, β_study area_, β_age class_, β_sex_, ρ_j_	834.3	0.051

During the decline study period and across study areas, other health was the leading cause of death (38.1%, 10.9%–67.2%), followed by *P. tenuis* (34.8%, 12.0%–61.2%), ticks (13.3%, 1.1%–34.8%), predation (7.4%, 0.4%–23.4%), and other (6.2%, 0.4%–20.4%). Our best CSM model was $\alpha_{k}$ *+ β*_death age class_ *×* death age class_*ik*_ (*w*_*i*_ = 0.302; Table 2). However, death age class did not have a statistically significant effect on CSM probabilities.

During the population stabilization study period and across study areas, predation was the leading cause of death (33.2%, 19.4%–48.2%), followed by other (21.7%, 11.2%–34.6%), other health (17.2%, 2.8%–35.8%), *P. tenuis* (15.7%, 5.0%–29.7%), and ticks (12.2%, 2.8%–27.5%). Our best CSM model was $\alpha_{k}$ + *β*_sex_ × sex_*ik*_ + *β*_death age class_ × death age class_*ik*_ (*w*_*i*_ = 0.596). However, sex and death age class did not have a statistically significant effect on CSM probabilities.

### 3.3 Survival

Baseline annual survival for the entire study period was 84.8% (81.3%–87.9%). Our best survival model was *γ + β*_study area_ *×* study area_*i*_ *+ β*_age class_[ageclass_*ij*_] *+ ρ*_*j*_ (*w*_*i*_ = 0.328). Neither covariate was statistically significant. Annual survival was 84.4% (80.36%–88.0%) in GPIR and 87.1% (69.7%–95.2%) in VNP. Overall female survival was 86.5% (CI = 82.5%–89.8%) and overall male survival was 79.1% (60.1%–90.1%).

Baseline annual survival during the decline period was 86.5% (80.6%–91.1%). Our best model was *γ + β*_study area_ × study area_*i*_ *+ ρ*_*j*_ (*w*_*i*_ = 0.443). The study area covariate was not statistically significant. Annual survival in GPIR was 83.3% (74.0%–90.2%) and 92.8% (74.0%–98.1%) in VNP. Overall, female survival was 87.6% (81.1%–92.4%) and overall male survival was 86.0% (53.4%–97.1%).

Baseline annual survival for the stabilization period was 85.6% (81.1%–89.5%). Our best model was *γ + β*_study area_ × study area_*i*_ *+ β*_age class_[ageclass_*ij*_] *+ ρ*_*j*_ (*w*_*i*_ = 0.324). Neither covariate was statistically significant. Annual survival in GPIR was 86.1% (81.5%–90.1%) and 66.2% (14.6%–91.7%) in VNP. Overall, female survival was 87.1% (2.3%–90.1%) and overall male survival was 79.1% (53.2%–90.7%).

### 3.4 Moose and Deer Density Estimates

**FIGURE 3:**
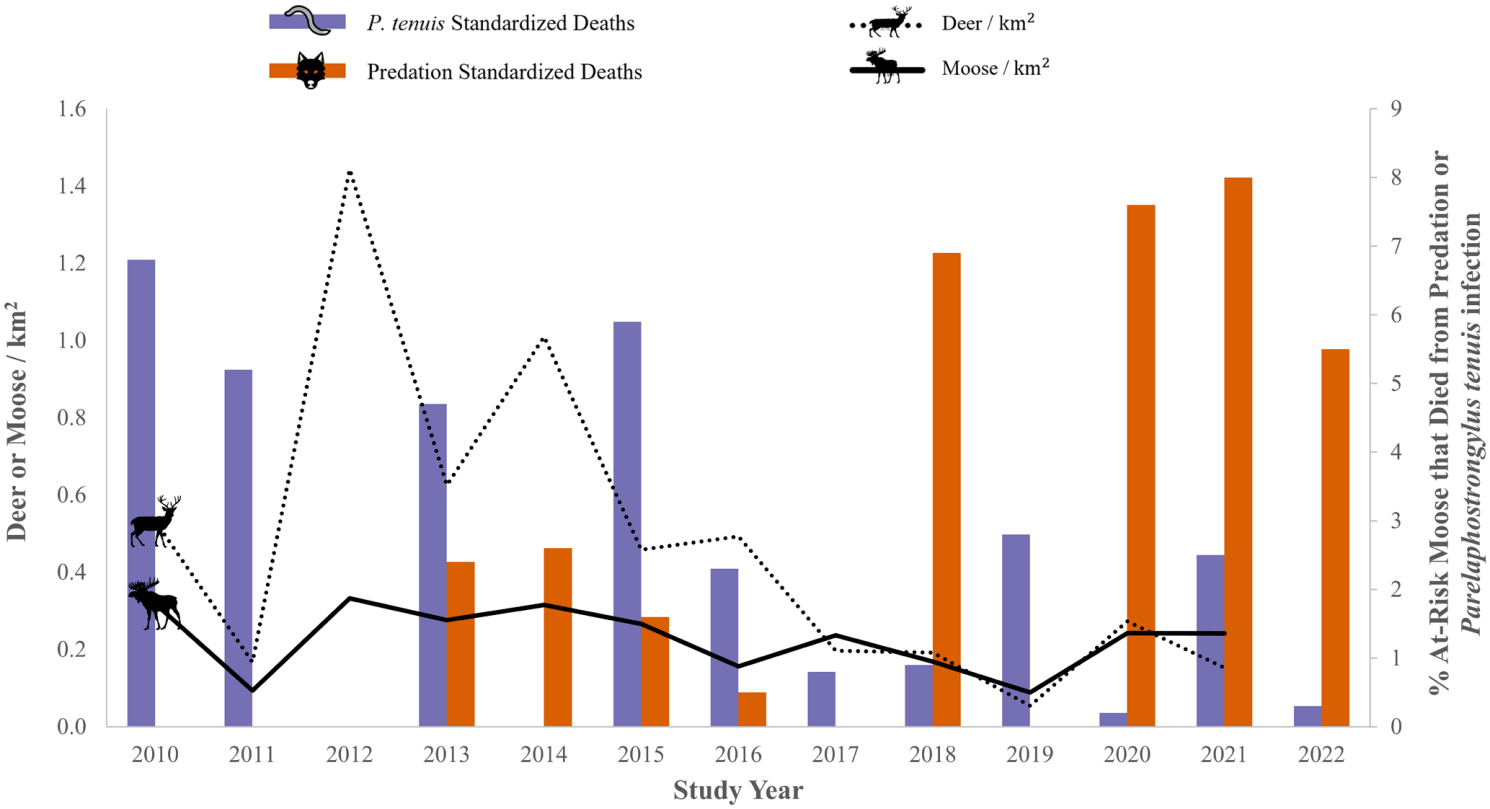
The percent of at‐risk moose that died from *Parelaphostrongylus tenuis* or predation each year overlayed with moose and deer densities on Grand Portage Indian Reservation (GPIR). Standardized deaths are the total proportion of mortality assigned to moose for a given cause in a year divided by the number of moose at risk that year. Deer densities are raw counts, whereas moose densities are corrected for sightability.

Moose densities in GPIR ranged from 0.09/km^2^ in 2011 and 2019 to 0.33/km^2^ 2012 (Figure 3). Deer counts in GPIR ranged between 0.05 (11 individuals, 2019) and 1.44 deer/km^2^ (293 individuals, 2012; Figure 3).

## 4 Discussion

Long‐term wildlife monitoring programs can capture population dynamics of vulnerable species critical to supporting Indigenous peoples' lifeways, and the findings of this study led by the Grand Portage Band of Chippewa are a testament to the value of Indigenous leadership in wildlife research and management. This study illustrates that *P. tenuis* ‐related mortality and predation probabilities for adult moose shifted drastically over 12 years. In the first 4 years, moose died primarily from *P. tenuis* or other health issues, and predation was relatively uncommon. Conversely, predation mortality probability nearly quadrupled in the last 8 years of the study while *P. tenuis* and other health issue probabilities were halved. This observation is pertinent to moose, deer, and wolf population management and would not have been captured without a long‐term collar‐monitoring program developed by an Indigenous nation. In addressing the specific causes of moose mortality identified in this research, the Grand Portage Band, governmental agencies, and universities will continue to enhance their co‐stewardship practices, ensuring that moose populations—and the cultural and subsistence practices they support—thrive well into the future.

Changes in deer density likely explain both the high *P. tenuis* ‐related moose mortality during the moose decline and the low *P. tenuis* ‐related mortality during the stabilization period. Because deer are *P. tenuis* 's definitive host, increased aberrant transmission to moose usually correlates with high deer densities (Lankester 2010). This expectation held in our study, as *P. tenuis* infection was most common when deer densities were highest in GPIR (~1.4 deer/km^2^; Figure 3). Conversely, northeastern Minnesota experienced an unusually severe winter in 2013–2014, causing a prolonged deer density decline (Michel and Giudice 2022). The deer density nadir in GPIR during our study (0.2 deer/km^2^) occurred simultaneously with lower *P. tenuis* ‐induced mortality in moose (Figure 3), and the low *P. tenuis* mortality in moose lasted for 9 years following the deer population decline as deer populations have remained low. While the deer densities in our study are not corrected for sightability, local biologists believe that they are near‐census counts given that deer are concentrated within 1 km of Lake Superior's shore at the time of the counts due to deep snow inland. Taken together, this suggests reducing deer densities is necessary to reduce *P. tenuis* transmission, and the threshold for reducing transmission may be far below the current recommendation of 4 deer/km^2^.

During the stabilization period of this study, the proportion of mortalities due to wolf predation quadrupled relative to the decline period and was our leading source of mortality (Figure 2). Wolves preying on deer may have played a role in keeping deer numbers low after the weather‐related decline, but high and stable wolf densities combined with fewer deer to sustain them (Erb and Humpal 2022) may have also prevented moose population recovery to pre‐decline numbers via an increased predation rate on moose. While our results cannot definitively tie predation of adults to the lack of population recovery, previous studies performed by the Grand Portage Band and collaborators in our study area suggest high predation rates on calves by both wolves and black bears during the same time period (70%–90% /year; Moore, Wolf et al. 2024; Chenaux‐Ibrahim et al. 2024; Van de Vuurst et al. 2022; Wolf et al. 2021; Severud et al. 2019). The Grand Portage Band also developed a spring black bear hunting season in 2016 to reduce neonate moose predation by bears, which reduced bear predation on moose calves by 68% in years when the hunt was held (Moore, Wolf, et al. 2024). That study suggests that such activities are of high value in conserving moose populations on lands under Indigenous co‐stewardship. Given the increased predation on adults and co‐occurring lack of moose population recovery observed in our study, efforts to translocate wolves or otherwise reduce predation on adult moose are worthy of consideration in tandem with deer population reduction efforts and other management actions aimed at reducing disease and improving habitat.

By understanding that the primary causes of moose mortality have transitioned from parasitism‐related factors to predation, the Grand Portage Band and other resource‐managing entities can optimize their co‐stewardship practices to address more than a single parasitic vector species or predator species that impacts moose and capture the dynamic and complex ecological relationships that influence moose survival. When early results from this study indicated that mortality by brainworm was high, the Grand Portage Natural Resources department maintained lower deer densities on reservation lands. The northeastern Minnesota deer population experienced a severe natural decline due to a period of two severe winters in a row (2013–2015). Following this natural decline in deer density, the Grand Portage Band initiated the following deer management strategies to maintain those low densities: (1) an early and late antlerless season (2016), (2) offered extra antlerless tags to non‐Tribal resident hunters (2016), (3) allowed up to five antlerless deer for direct descendants of the Grand Portage Band (2016), and (4) added an archery season for non‐tribal resident hunters (2017). Since these strategies were initiated, the proportion of antlerless deer harvested grew from < 10% of deer harvest to > 50% of total harvest. Except for the early antlerless season, these management strategies remain in place and contribute to holding the deer population at low levels. In turn, this appears to have driven the lower *P. tenuis* infection rates in adult moose (Figure 3). Similarly, to address rising predation, the Grand Portage Band translocated several wolves to Isle Royale National Park (Verant et al. 2022; Hervey et al. 2021; Romanski et al. 2020; Orning et al. 2020). Taking this holistic approach to management that also considers not only continuing to monitor and manage deer populations but also exploring strategies to mitigate wolf predation on moose, especially in years when *P. tenuis* ‐induced mortality is low, aligns with the Band's stewardship principles and ensures that both health‐related issues and predation pressures are addressed.

Our finding that *P. tenuis* , predation, and other health issues are key adult moose mortality sources in Minnesota aligns with previous studies, and our CSM analysis framework provides more clarity for directing future management action. Carstensen et al. (2017) reported that primary causes of death were predation (32%), parasites (30%), and bacterial infections (21%) among 57 mortalities of collared adult moose. However, eight mortalities assigned to predation also involved predisposing health issues, four assigned to bacterial infections were associated with predation attempts, and one assigned to bacterial infection was associated with *P. tenuis* infection. Therefore, CSM point estimates varied depending upon perspective and remained unclear. By assigning individual cause of death proportion predictive probabilities via Bayesian priors, we provide a population‐level point estimate that explicitly incorporates the multifactorial nature of adult moose deaths. Our approach has been shown to improve inference about the “true” relative strength of a cause of mortality in a population (Walsh et al. 2018).

Our estimates of the relative contributions of wolves and health‐related issues to adult moose mortality remain imperfect but are an improvement on traditional methods used to drive management actions. Similarly to Carstensen et al. (2017), there were five cases where we could not examine wolf‐killed animals for health issues because the carcass was consumed. In these cases, the summary of the evidence (including kill site and GPS data) suggested wolf kills, but given that few samples remained for testing for *P. tenuis* or other health issues, it is possible that we overestimated the contribution of predation and underestimated health‐related issues. To illustrate the potential magnitude of bias, if we evenly assigned the probability of cause of death to each category in those 5 cases during the stabilization period (when all these mortalities occurred), predation would remain a more common cause of mortality than other health and *P. tenuis* (23% vs. 22% and 17%, respectively). This suggests that predation was a primary source of mortality during the stabilization period even when potential bias against detecting health issues is taken into account.

That adult moose survival was relatively stable across the decline and stabilization periods (~86%) is surprising given that population growth rates in Minnesota are most sensitive to changes in adult survival (Severud et al. 2019). However, when broken down by study area, survival did increase in GPIR (from 83% to 86%), whereas it drastically decreased in VNP (from 93% to 66%). The significant decrease in our calculated VNP survival is likely an artifact of sampling bias because so few animals were followed in VNP during the stabilization period (9 in VNP vs. 131 in GPIR; Table 1) and new collars were not deployed in VNP after 2012. Because moose were collared continuously throughout the study in GPIR, the survival rate and trend in that area may be more indicative of moose adult survival rates in northeastern Minnesota and align with our expectation that adult survival should have increased from the decline to the stabilization period.

Even when considering GPIR's estimated adult moose survival rate alone, it is lower than thriving populations (~89%; Franzmann and Schwartz 2007), but not abysmal. Because moose are semi‐aquatic, several of the collars that were right‐censored because they stopped working were likely mortalities where the GPS signal was obstructed due to being underwater. Thus, our survival estimates may be biased slightly high. An alternative explanation for the decline is poor calf recruitment, which usually varies more widely and frequently than adult survival and, therefore, can also have a strong effect on population growth trajectories (Severud et al. 2019). The analyzed dataset does not contain calves, so we did not empirically assess this here. However, data from several related moose studies in this ecosystem, including those previously published from this study group, indicate that recruitment rates can be highly stochastic and occasionally quite low (~10%), making recruitment a more impactful parameter on population growth than previously thought (Moore, Wolf et al. 2024; Chenaux‐Ibrahim et al. 2024; Van de Vuurst et al. 2022; Wolf et al. 2021). We, therefore, posit that low recruitment may be driving the lack of population recovery despite adult survival rates that appear ecologically healthy.

Indigenous nations are working to sustain native subsistence species vital to their lifeways through climate vulnerability assessments and adaptation planning (Hessami et al. 2021; Stults et al. 2016; Moore, Wolf, and Travis 2015), and long‐term, Indigenous‐led collaring programs are integral tools in these efforts. In the case of our study, a long‐term program enabled the novel insight that *P. tenuis* was the primary mortality factor during the population decline period, followed by a rise in predation during the stabilization period. Because climate change is predicted to increase the frequency of stochastic weather events such as that which caused the deer decline in our study (Pörtner et al. 2022; Trenberth 2011), short‐term collaring projects may no longer provide enough data to adequately and adaptively inform management. Similarly, long‐term monitoring data give Indigenous communities a strong foundation from which to advocate for holistic stewardship of native subsistence species. The monitoring program that produced the data presented in this paper is a vital tool in advocating for increased deer harvest in the 1854 Ceded Territory, where deer harvest quotas and seasons for non‐tribal hunters are set by the Minnesota Department of Natural Resources. A strong constituency of state‐licensed deer hunters, whose license purchases pay for many of the Minnesota DNR's activities, advocates through paid lobbyists for higher deer densities in core moose range than are good for moose. Hence, despite key Great Lakes Chippewa natural resource decision makers serving on the deer management board for the Minnesota Department of Natural Resources, the department is under considerable financial and political pressure to ignore the Tribal recommendations to manage deer at low population densities. To continue to apply their own pressure to maintain low deer densities in core moose range, the Grand Portage Band has used this long‐term monitoring data in consultations with the state, in mainstream media pieces, presentations with the public, and peer‐reviewed literature recommending holistic management to restore moose (Moore, Severud, et al. 2024; Moore, Wolf, et al. 2024). The rewards of long‐term wildlife collaring and monitoring efforts, therefore, justify the additional cost and time commitment, especially for species of high intrinsic and cultural value to Indigenous people. By continuing to integrate long‐term monitoring programs into their co‐stewardship practices, Indigenous stewards can further strengthen their role as guardians of the land, ensuring that moose populations remain stable and resilient for future generations.

The implications of this study extend beyond immediate management actions. By demonstrating a successful collaboration between Indigenous, federal, and university researchers that also integrates indigenously‐ and federally collected datasets, it contributes to the broader framework of Indigenous co‐stewardship (Moore, Severud, et al. 2024; Moore, Wolf, et al. 2024). Studying the drivers of CSM of moose on these lands is not simply a matter of ecological importance, nor an academic nor theoretical exercise; it is a testament to the resilience and continuity of Indigenous stewardship practices and empowers the Grand Portage Band to make informed decisions that are rooted in both traditional ecological knowledge and contemporary science. The ability of the Grand Portage Band to lead and direct research on their own terms, focusing on species that are critical to their culture and subsistence, sets a powerful precedent for other Indigenous nations. It is an exercise of sovereignty and a necessary step to ensure that their lifeways, and those of future generations, are preserved. By leading this work, the Grand Portage Band is demonstrating rights to self‐determination in stewarding their natural resources and also contributing valuable knowledge to the broader field of ecology and conservation. This research model not only respects Indigenous sovereignty but also enriches the overall understanding of wildlife ecology in the context of changing environmental conditions.

## Author Contributions

**Tyler J. Garwood:** conceptualization (equal), data curation (lead), formal analysis (lead), investigation (supporting), methodology (equal), validation (equal), visualization (lead), writing – original draft (lead), writing – review and editing (lead). **William J. Severud:** conceptualization (equal), data curation (lead), formal analysis (supporting), investigation (supporting), methodology (equal), writing – original draft (equal), writing – review and editing (equal). **Steve K. Windels:** conceptualization (equal), data curation (supporting), funding acquisition (lead), investigation (lead), project administration (lead), resources (lead), supervision (equal), writing – review and editing (equal). **Arno Wünschmann:** data curation (equal), investigation (lead), methodology (equal), writing – review and editing (equal). **Edmund J. Isaac:** conceptualization (supporting), data curation (supporting), funding acquisition (supporting), investigation (lead), project administration (equal), writing – review and editing (equal). **Anibal G. Armien:** data curation (equal), investigation (equal), methodology (equal), writing – review and editing (equal). **Seth A. Moore:** conceptualization (lead), data curation (equal), formal analysis (supporting), funding acquisition (lead), investigation (lead), methodology (equal), project administration (lead), resources (lead), supervision (lead), writing – original draft (supporting), writing – review and editing (equal). **Tiffany M. Wolf:** conceptualization (lead), data curation (equal), formal analysis (supporting), funding acquisition (lead), investigation (equal), methodology (equal), project administration (lead), resources (equal), supervision (lead), validation (supporting), writing – original draft (supporting), writing – review and editing (equal).

## Conflicts of Interest

The authors declare no conflicts of interest.

## Supporting information


Table S1.

## Data Availability

The Grand Portage Band of Lake Superior Chippewa, a sovereign Indigenous nation, served as a primary intellectual and financial driver of this manuscript and is unwilling to share these data publicly on the basis of Indigenous data sovereignty. However, much of the data necessary for performing the analysis for this study, as well as further details on data collection, are available in the Data S1 of this article. The Grand Portage Band also offers that interested parties may request permission to access more of the data by contacting senior authors and entering a data ownership and sharing agreement. In maintaining ownership of these data, the Band ensures that any future western scientific products that emerge from these data integrate and reflect their cultural priorities.
